# Supplementary material for: Inflammatory factors and risk of lung adenocarcinoma: a Mendelian randomization study mediated by blood metabolites
Source: Front Endocrinol (Lausanne). 2024 Aug 27;15:1446863. doi: 10.3389/fendo.2024.1446863 (PMC11384989; doi:10.3389/fendo.2024.1446863)
Supplement: Supplementary file 1 [file DataSheet1.zip › supplementary files/S2/GCST90200853/sensitivity-analysis.pdf]

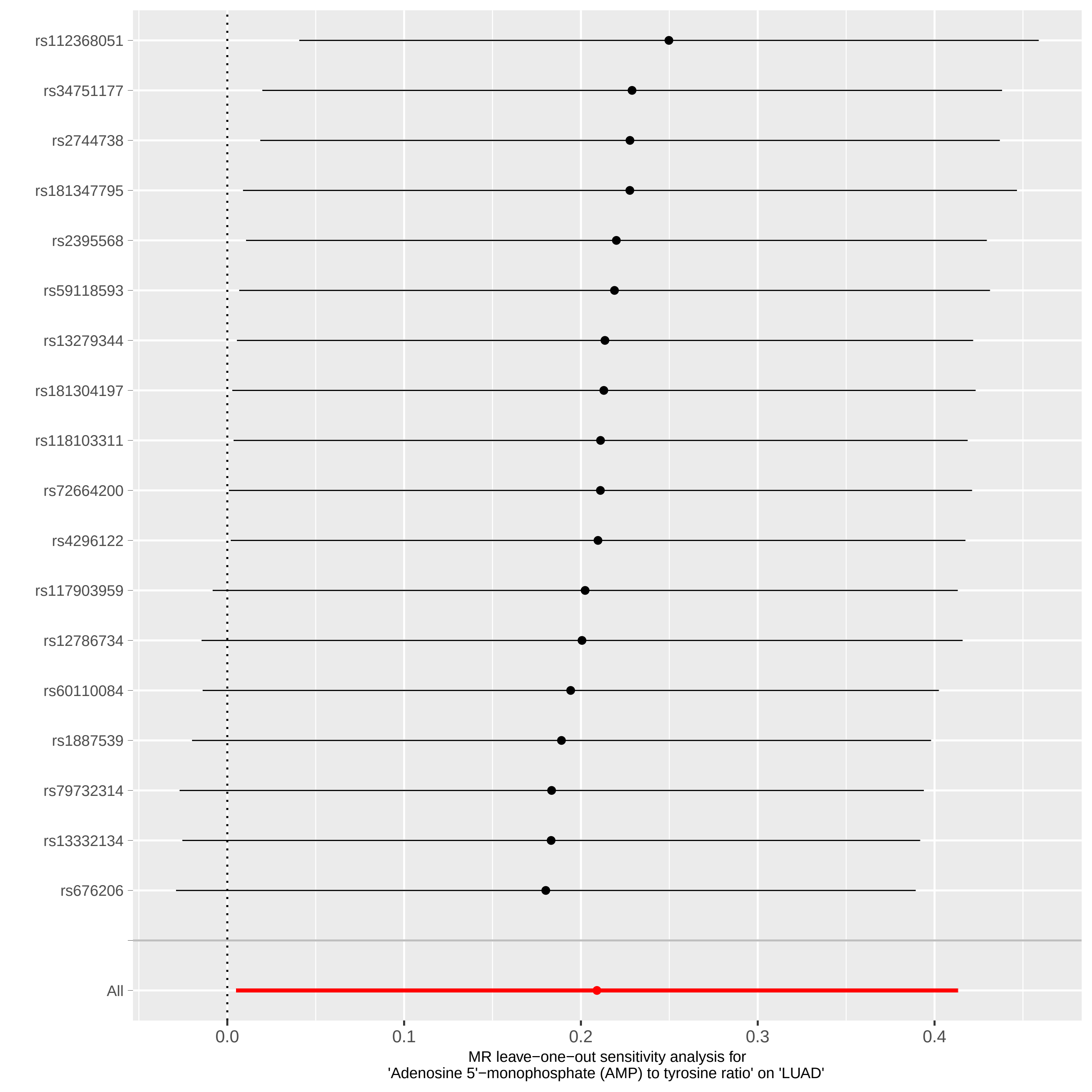
rs112368051
rs34751177
rs2744738
rs181347795
rs2395568
rs59118593
rs13279344
rs181304197
rs118103311
rs72664200
rs4296122
rs117903959
rs12786734
rs60110084
rs1887539
rs79732314
rs13332134
rs676206
All
0.0
0.1
0.2
0.3
0.4
MR leave−one−out sensitivity analysis for
'Adenosine 5'−monophosphate (AMP) to tyrosine ratio' on 'LUAD'
